# Air protection programmes in Poland in the context of the low emission

**DOI:** 10.1007/s11356-017-9233-9

**Published:** 2017-05-25

**Authors:** Janusz Adamczyk, Arkadiusz Piwowar, Maciej Dzikuć

**Affiliations:** 1 Faculty of Economics and Management, University of Zielona Góra, ul. Licealna 9, 65-417 Zielona Góra, Poland; 2 Faculty of Engineering and Economics, Wrocław University of Economics, Komandorska Street118/120, 53-345 Wrocław, Poland

**Keywords:** “Low emission”, Air protection programmes, LCA analysis, Choice of heat

## Abstract

The protection of the air against pollutants from individual boiler plants is a big challenge in Poland. It results mainly from the preference for coal, the national energy carrier, the use of old low-efficiency boilers and the location of Poland in a temperate climate where the heating period lasts at least 5 months. This article presents a wide range of activities aimed at the reduction of the environmental impact of the emissions of pollutants from individual heat sources—the so-called low emission. The article presents the extent of the national legislation resulting from the European Union regulations. It discusses the assumptions of the air protection programmes (APPs) and the low emission reduction programmes (LERPs). The assumptions mentioned above are analysed as part of a life cycle assessment (LCA) analysis and a multi-criterion analysis. An important result of these analyses (in the Polish conditions) is the conclusion that a boiler fired with large pieces of wood is an optimal solution from the economic and ecological points of view. The article proposes systemic, organisational and legislative solutions whose implementation could contribute to raising the effectiveness of the protection of the atmosphere.

## Introduction

The combustion of fuels for the purposes of central heating and domestic hot water preparation in buildings causes emissions of gaseous and particulate matter pollutants into the air, considered to be one of the key factors affecting the state of the natural environment (Proszak-Miąsik et al. 2013). This negative impact is reduced primarily by optimising the consumption of energy in buildings, for example through the application of thermal insulation in buildings and heat pipes or the replacement of old boilers. The costs of such projects are high and often exceed investors’ financing capabilities. This has contributed to the introduction of a number of tools to give preferential financial support to environmental protection projects in Poland, including those in the field of the protection of the atmosphere. The introduction of the instruments for the support of the protection of the atmosphere in Poland has not been historically intended to provide economic support only, but has also been the result of Poland’s commitments to improve environmental conditions. Financing environmental activities is intended to accelerate the process of changing and serving as an incentive for investors.

Most of the financial instruments are directed at businesses, local government units and other institutional players. Meanwhile, it is a well-known fact that air quality problems are resulted largely from the combustion of fuels for heating purposes in the individual boiler plants installed in residential buildings—the so-called low emission,^1^ which is the main cause of the non-observance of the air quality standards specified by the EU legislation (Directive 2008/50/EC; Directive 2004/107/EC). The low emission problem is exacerbated in Poland by the lack of legal instruments relating to individual buildings, the enforcement of which would make it possible to control the emission levels. Legal instruments are used only in relation to production plants and public buildings. It can be expected that this state of affairs does not motivate residential building owners, managers and tenants to take any actions to minimise their environmental impact. The small number of financial instruments available to individual households in Poland is the cause of the low interest in the activities aimed at the improvement of the energy efficiency. The financing sources for individual households are described in the low emission reduction programmes (LERPs). The LERP will be discussed later in this article.

It should be noted that Poland, Romania and Germany are the EU countries where emissions of benzo(a)pyrene (B(a)P) have increased over the last decade. The overall emission level for this gas in all the EU countries (between 2004 and 2013) remained unchanged (*Air quality in Europe* 2015; *Jakość powietrza w Polsce* 2015). In 2013, 87% of the EU’s urban population were exposed to PM_2.5_ concentrations that exceeded the values established by the WHO^2^ as the limit levels for the protection of human health. The exposure to ozone in urban areas is still very high. In 2013, 98% of the EU citizens were exposed to O_3_ concentrations that exceeded the limits recommended by the WHO (*Air quality in Europe* 2015). The increased emission of pollutants into the atmosphere in Polish cities does not result only from the combustion of fuels for the production of thermal energy in buildings—other factors include transportation, waste management and manufacturing. However, the low emission (the emission from individual boilers) is one of the major problems affecting the state of the atmosphere in Poland.

The combustion of fuels in the low-power combustion sources causes emissions of substances, such as particulate matter (164,000 Mg^3^), nitrogen oxides (93,000^3^ Mg), sulphur dioxide (284,000^3^ Mg), carbon monoxide (1843,000^3^ Mg) and heavy metals (1000^3^ Mg) (GUS 2015). For comparison, the total emission of the main air pollutants^4^ in the same year (2013) was 407.000 Mg for particulate matter, 799,000 Mg for nitrogen oxides, 847,000 Mg for sulphur dioxide, 2,876,000 Mg for carbon monoxide and 2800 Mg for heavy metals (GUS 2015).

Table 1 presents the percentage share of individual energy carriers used in households for heating in Poland in 2012.

**Table 1:** The percentage share of individual energy carriers used in households for heating in Poland in 2012 [%]

Network heat	Hard coal	Biomass (wood)	Methane-rich natural gas	Electricity	Lignite	Nitrogen-rich natural gas	Coke	Fuel oil	Liquid gas	Other
41.54	40.84	39.98	8.83	5.35	1.36	0.94	0.74	0.45	0.32	4.45

Source: *Energy consumption* 2014

It should be noted that the values presented in the table apply to all households, including single-family and multi-family buildings. Network heat is rarely used in Poland to heat single-family houses, mostly due to the high investment costs (one linear metre of network connection costs around 400 PLN^5^). Therefore, it is mostly people living in multi-family blocks located in cities that have access to the cheaper and more environmentally friendly urban heat, and network heat solutions are rarely used in suburban buildings.

The popularity of using solid fuel boilers in Poland results primarily from the low cost of obtaining heat. Table 2 shows the mean prices of heat produced from different types of fuels in Poland in 2012.

**Table 2:** Arithmetic mean prices of heat produced from different types of fuels in Poland in 2012 [PLN/GJ]

Liquid gas	Fuel oil	Nitrogen-rich natural gas	Methane-rich natural gas	Network heat	Pellets (biomass)	Coke	Hard coal	Lignite	Wood (biomass)
105.7	105.0	88.3	71.1	47.3	35.0	32.8	28.3	28.2	12.8

Source: *Energy consumption* 2014

The heating cost for hard coal is one of the lowest among those presented for individual households. The lowest heating cost is that of wood (biomass), but this type of energy carrier is not very popular in Poland, among others due to higher frequencies of boiler operation. The heat obtained from biomass—pellets—is nearly three times as expensive but eliminates the nuisance of having to operate the boiler at more frequent rates. The highest costs are generated by heating with oil and liquid gas. The network heat cost, in turn, is more or less at the average level. The cost of producing heat from sources considered to be more environmentally friendly is high in Poland; this does not apply to burning wood (biomass).

Mostly coal with low heating parameters was used in Poland—imported until 2015 from Russia. However, in 2015 an amendment to the Act of 25 August 2006 on the fuel quality monitoring and control system (Dz. U. of 2015, item 1361) was passed to prevent this from happening. The amendment introduced quality standards for solid fuels—coal and its derivatives. The Customs Service was tasked with ensuring that only coal of a specific quality may enter the Polish market, and the Trade Inspection Agency was made responsible for its trade control (Ustawa z dnia 25 sierpnia 2006).

Another problem that contributes to the increased emissions is the technical condition of boilers, which does not meet the technical requirements. The inspection of the condition of central heating boilers in individual buildings is limited, as well. It is used rarely only for the purposes of the energy certification when a building is being sold or rented. Central heating boilers have relatively low efficiency, which negatively affects the combustion processes and also contributes to the increased emissions. According to the statistical data, the average life of a solid fuel boiler in Poland is 10.3 years (*Energy consumption* 2014).

The aim of this article is to present the atmosphere protection system in the context of the reduction of the low emission impact in Poland. The article discusses the importance of the air protection programmes (APPs) and the low emission reduction programmes (LERPs) in the context of their importance to the protection of the air. The main methodological assumptions applicable to the above-mentioned programmes were subjected to a life cycle assessment (LCA) analysis, and some legal considerations of the system were presented.

## Legal status

### Legal considerations related to the minimisation of the impact of the low emission in Poland

The Act of 13 April 2012 amending the Environmental Protection Law Act (Dz. U. of 2012, item 460) transposes into the Polish law the provisions of the Directive 2008/50/EC of the European Parliament and of the Council of 21 May 2008 on ambient air quality and cleaner air for Europe (OJ L 152, 11 June 2008, p. 1). Directive 2008/50/EC of the European Parliament and of the Council was established as a result of the implementation of the thematic strategy on air protection, based on “*the sixth Community action programme on the environment*”, whose targets included e.g. the reduction of air pollution down to the levels that minimise their harmful effects on the health of the public and its most vulnerable groups, in particular. The directive specifies the air pollution levels together with the dates of their application and introduces new air quality management mechanisms to be used in zones and agglomerations.

In Poland, there are at least a few legal acts in force that regulate the state of the atmosphere. One of the acts is the above-mentioned Act of 25 August 2006 on the fuel quality monitoring and control system. Article 3a thereof provides that solid fuels placed on the market shall comply with the quality requirements specified for this type of fuel for the purpose of environmental protection, the impact on human health and the interests of consumers (Ustawa z dnia 25 sierpnia 2006). The main weakness of this act is that no regulation on the quality requirements for solid fuels has been published yet. Although the act specifies penalties for the trade in low-quality coal, this practice may continue until the guidelines are published under a regulation of the minister responsible for the economy.

The superior act on the environmental management in Poland is the above-mentioned Environmental Protection Law (Dz. U. of 2008, No. 25, item 150, as amended). In accordance with Article 91 thereof, “*an air protection programme must be developed for zones* 6 *where exceedances of the limit levels or target levels, where applicable plus the margin of tolerance, were found for at least one substance*”, out of the substances set out in the regulation of the Minister of Environment as of 24 August 2012 on the levels of certain substances in the air (*Rozporządzenie Ministra Środowiska z dnia 24 sierpnia* 2012).

The annual air quality assessment is performed for the substances for which the normative values of concentration in the air (limit levels/target levels/long-term objectives) are specified in the national law and EU directives.

The assessment is performed based on the criteria set out for *Rozporządzenie Ministra Środowiska z dnia 24 sierpnia* (2012):

- the protection of human health
- the protection of plants

The assessment of the fulfilment of the criteria set out for the protection of human health takes into account 12 substances (*Rozporządzenie Ministra Środowiska z dnia 24 sierpnia* 2012):

- sulphur dioxide SO_2_
- nitrogen dioxide NO_2_
- carbon monoxide CO
- benzene, C_6_H_6_
- ozone O_3_
- particulate matter PM_10_
- lead Pb in PM_10_
- arsenic As in PM_10_
- cadmium Cd in PM_10_
- nickel Ni in PM_10_
- benzo(a)pyrene B(a)P in PM_10_
- particulate matter PM_2.5_

The assessments of the fulfilment of the criteria set out for the purpose of protection of plants take into account three substances (*Rozporządzenie Ministra Środowiska z dnia 24 sierpnia* 2012):

- sulphur dioxide SO_2_
- nitrogen oxides NO_*x*_
- ozone O_3_

In addition, Directive 2008/50/EC of the European Parliament and of the Council of 21 May 2008 on ambient air quality and cleaner air for Europe provides that in the event of exceedances of those limit values for which the attainment deadline is already expired, the air quality plans (called programmes in the *Environmental Protection Law* Act) shall set out appropriate measures, so that the exceedance period can be kept as short as possible (Directive 2008/50/EC).

Air quality assessments in a given zone are made in Poland by the Regional Inspector of Environmental Protection^7^ based on the conducted air quality monitoring. It forms the basis for the classification of zones.^8^ The classification of a zone into class C means that an air protection programme (APP) needs to be developed.^9^ Tables 3, 4 and 5 show zone classes and the required activities depending on the pollutant concentration levels.

**Table 3:** Zone classes and activities required depending on the pollution levels determined as part of the annual air quality assessment for cases where a limit level is specified and no margin of tolerance^a^ is specified for the pollutant

Class zone	Pollutant concentration level	Activities required
A	Foes not exceed the limit level^b^	• Maintain the pollutant concentrations below the limit level and strive to maintain the best ambient air quality in conformity with sustainable development.
C	Above the limit level^b^	• Specify the areas where limit levels are exceeded. • Develop or update the air protection programme to achieve appropriate limit levels for substances in the air. • Control pollutant concentrations in the areas where limit levels were exceeded and conduct activities to decrease the concentrations at least to the limit level values.

^a^This applies to the following pollutants: sulphur dioxide SO_2_, nitrogen oxide NO_2_, carbon oxide CO, benzene C_6_H_6_, particulate matter PM_10_ and lead Pb in PM_10_—health protection; and sulphur dioxide SO_2_, nitrogen oxides NO_*x*_—plant protection

^b^Taking into account the allowable frequencies for exceedances referred to in the regulation of the Minister of Environment on the levels of certain substances in the air

**Table 4:** Zone classes and activities required depending on the pollution levels determined as part of the annual air quality assessment for cases where the limit level and the margin of tolerance are specified for the pollutant

Class zone	Pollutant concentration level	Activities required
A	Does not exceed the limit level	• Maintain the pollutant concentrations below the limit level and strive to maintain the best ambient air quality in conformity with sustainable development.
B	Above the limit level but does not exceed the limit level plus the margins of tolerance	• Specify the areas where the limit level is exceeded. • Determine the reasons for the exceedance of the limit level for the substance in the air, and take action to reduce emissions of the substance.
C	Above the limit value plus the margin of tolerance	• Determine the areas where the limit value and the limit value plus the margin of tolerance are exceeded. • Develop or update the air protection programme aimed at achieving the limit values for substances in the air.

**Table 5:** Zone classes and activities expected depending on the pollutant concentration levels determined as part of the annual air quality assessment for cases where the target value^a^ is specified for the pollutant

Class zone	Pollutant concentration level	Activities required
A	Does not exceed the limit value^b^	• None
C	Above the limit value^b^	• Strive to achieve the target value for the substance within a specified time using economically reasonable technical and technological measures. • Develop or update the air protection programme to achieve appropriate target values for substances in the air.

^a^Applies to ozone O3 (human health protection, plant protection) and arsenic As, cadmium Cd, nickel Ni, benzo(a)pyrene B(a)P in PM_10_—human health protection. The target value is also an extra parameter to be taken into account in the annual air quality assessment for particulate matter PM_2.5_. The limit value is the primary criterion for the assessment of PM_2.5_

^b^Taking into account the acceptable frequencies for exceedances specified in the regulation of the Ministry of Environment on the levels of certain substances in the air

The Regulation *on the assessment of the levels of substances in the air* specifies the following (*Rozporządzenie Ministra Środowiska z dnia 13 września* 2012a):

- the methods and scope of the assessment of substance levels in the air
- the upper and lower assessment thresholds for certain substances in the air and the acceptable frequencies for exceedances of the assessment thresholds
- the scopes of the required measurements, including the division into continuous and periodic measurements
- the criteria for the location of sampling points
- the minimum number of fixed sampling points
- etc.

The subsequent Regulation of the Minister of Environment further specified how to calculate the average exposure indicators and assess the fulfilment of the exposure concentration obligation. This Regulation specifies how to (*Rozporządzenie Ministra Środowiska z dnia 13 września* 2010b)

- calculate the average exposure indicator values for a city with a population of more than 100,000 people and agglomerations
- calculate the value of the national average exposure indicator
- assess the fulfilment of the exposure concentration obligation

In accordance with the Regulation of the Minister of Environment of 10 September 2012 *on the scope and method of transmission of information on air pollution*, for each class C zone for a specific substance, information is given on exceedances of appropriate criterion values for concentrations of this substance (*Rozporządzenie Ministra Środowiska z dnia 10 września* 2012):

- recorded on the basis of the measurements
- determined on the basis of other assessment methods

Exceedances are reported for each substance and position where the critical concentrations were exceeded with the frequency higher than permitted. The list of exceedances should include dates, concentration values and the cause of the exceedance. Similarly, in accordance with the applicable regulations, the main and other causes of the exceedance in the zone are determined.

It was assumed that the purpose of the air protection programme is to identify the directions of the corrective actions that will help achieve limit values or target values, specified from the viewpoint of the protection of human health and the environment within zone areas where these values are not met. APP should include a reference to

- the diagnosis of poor air quality on the basis of the analysis of the performed measurements and emission source inventories
- sources of emissions responsible for the air quality in the area of the zone and their percentage shares
- the estimated area where exceedances of the normative concentrations occur
- the number of people exposed to the pollutants
- the corrective actions intended to improve the cleanliness of the air together with the determination of specific objectives, tasks, their estimated costs and sources of financing and expected environmental effects

The national exposure reduction target to be met until 1 January 2020 for the concentration of particulate matter PM_2.5_ in the air is 18 μg/m^3^ and is specified in another regulation *on the national exposure reduction target* (*Rozporządzenie Ministra Środowiska z dnia 14 sierpnia* 2012).

Another regulation of 11 September 2012 *on air protection programmes and short-term action plans* (Dz. U. of 2012, item 1028) specifies the following (*Rozporządzenie Ministra Środowiska z dnia 11 września* 2012)

- detailed requirements that the air protection programmes and short-term action plans should meet
- the form of preparation and the necessary components of the air protection programmes and short-term action plans
- the scope of issues that should be identified and assessed in the air protection programmes and short-term action plans

### The nature of air protection programmes

The basic problem related to the quality of the air in Poland is the failure to meet the limit on the number of days with exceedances of the maximum daily mean level for particulate matter PM_10_ and the maximum annual mean level for particulate matter PM_10_, the maximum annual mean level for particulate matter PM_2.5_ and the exceedance of the benzo(a)pyrene target value. The low emission originating primarily from the municipal and residential sector, which includes individual sources of heat generation and hot water preparation, as well as small heating plants and transport are responsible for the state of the air quality in Poland.

The first air protection programmes were created in Poland in 2003 and 2004. In the period between 2003 and 2006, 161 zones were qualified to prepare APPs, with the total number of zones in the country being 362 (approx. 100 of them due to an exceedance of the PM_10_ limit values). In 2006, a new zoning was introduced. Compared to the previous years, their number changed from 362 to 170 and larger areas were created (from 2007, the annual air quality assessments were carried out in accordance with the new zoning). In 2008, 65 zones—that is 38% of all zones in the country—were qualified to prepare APPs. In 2009, approx. 100 zones were qualified to develop air protection programmes due to exceedances of the limit values for PM_10_ concentrations and the target values for benzo(a)pyrene. In most cases, target values for benzo(a)pyrene were exceeded in the zones that qualified for the APP due to an exceedance of the PM_10_ limit values. This does not mean that the air quality situation in Poland has improved, but rather the number of zones has decreased and the surface area of the zones covered by the air protection programmes increased considerably.

In 2010, the number of zones decreased again to 46 (this division is currently in force (*Rozporządzenie Ministra Środowiska z dnia 2 sierpnia* 2012)), 45 of which qualified for the preparation of the APP due to exceedances of limit values and target values for particulate matter PM_10_, particulate matter PM_2.5_, benzene, benzo(a)pyrene, nitrogen dioxide and arsenic dioxide. Between 2003 and 2010, APPs were developed for all the provinces, mainly as a result of exceedances of the limit values for particulate matter PM_10_ and benzo(a)pyrene.

The basic conclusions of the air protection programmes are as follows (Schönfelder 2010):

- Most programmes were developed due to exceedance of PM_10_ limit values and benzo(a)pyrene target values.
- Substances from the emission sources related to the municipal and residential sectors, i.e. the low emission, have the largest share of emissions of all the substances.
- The largest share in emissions is taken by surface sources (approx. 70% on average), linear sources (13%) and spot sources (17%) within the areas of various zones.
- The largest number of exceedances of the measured substances is recorded in Lesser Poland, Lower Silesian, Silesian and Kuyavian Pomeranian Provinces.

In accordance with the report (*Ocena jakości powietrza w strefach w Polsce* 2014), exceedances of PM_10_ limit values were recorded in 36 zones and exceedances of the PM_2.5_ limit value plus the margin of tolerance were recorded in 24 zones out of the total of 46 zones in the country. In addition, the failure of meeting the limit value for nitrogen dioxide was recorded in 4 zones. At the same time, exceedances of the target values were reported in 42 zones for B(a)P, in 4 zones for arsenic and in 6 zones for ozone.

In accordance with the report above, the individual building heating is responsible for the exceedance

- of the limit value for particulate matter PM_10_ (normative annual mean value 40 μg/m^3^) in 88.21% at the national scale
- of the limit value for particulate matter PM_2.5_ (normative annual mean value 25 μg/m^3^) in 86.5% at the national scale
- of the target value for B(a)P (normative annual mean value 1 ng/m^3^) in 98% at the national scale

Measurements of the B(a)P concentrations in Poland have shown exceedances of normative values for concentrations of this pollutant in a significant number of locations in the country for many years. The exceedances of the target value are associated with a significant increase in the concentrations of B(a)P in the winter (*Krajowy program ochrony powietrza* 2015).

The atmosphere protection tool in the form of the air protection programme was analysed for technical requirements. The analysis shows that programmes (*Krajowy program ochrony powietrza* 2015)

- do not contain a well-developed section on corrective actions
- do not include complete calculations of the estimated total cost of the corrective actions
- contain an incomplete diagnosis of the air quality in the indicated area and a non-exhaustive inventory of the emission sources
- indicate only those activities for which it is possible to obtain funding at the municipal level
- do not specify the correct method of monitoring the implementation of the corrective action, e.g. delays occur in the implementation of these actions
- indicate the low emission reduction programmes (LERPs) as major corrective actions despite those programmes having no statutory legal basis

LERPs are a bottom-up initiative based on the air protection programme or the municipal environmental protection programme. LERPs include a system for financing investment costs for the replacement of the heating system specified mostly in the form of a resolution of the city/municipality council (less often in the form of a city mayor order). It may take various organisational forms: from the simplest—financing occurs on the basis of an invoice through agreements concluded with residents (which limit the possibility of replacing the heating fuel back to solid fuel)—to quite complex programmes. The programmes adopt financing primarily for the purpose of the replacement of the heating system from a solid fuel to gas, oil or electric heating or a connection to the heating network.

## Method and results

### Life cycle assessment analysis of the assumptions of the low emission reduction programmes in Poland

As previously mentioned, the state of atmospheric air in Polish cities is devastating. The city of Kraków ranked the third in the list of the most polluted cities in the European Union in 2013 (*European Environment Agency AirBase database* 2016). As early as in November 2013, the authorities of Kraków introduced a ban on burning solid fuels in households—primarily coal—which will remain in effect until September 2018 (Adamkiewicz and Huscher 2014). In 2010, the diseases of the respiratory and circulatory systems, which are closely related to air pollution, contributed to the deaths of 507 people out of 100,000, which equals 52% of the total number of deaths in Poland (Wojtyniak et al. 2012).

The low emission reduction programmes (LERP) are implementation programmes of *the Environmental Protection Programmes*, whose adoption belongs to the responsibilities of the municipalities and districts, or *the Air Protection Programmes*. The LERP programmes present the funding methodology, the substantive part and the general assumptions.

*The Environmental Protection Programmes in Municipalities* are developed on the basis of

- the Environmental Protection Law
- the Energy Law
- the European Union legislation

It is believed that the implementation of the LERP programmes at the lowest levels of the administrative division^10^ of the country should contribute to the improvement of the atmosphere.

It was assumed that the LERP programmes would be implemented primarily by

1. replacing low-performance and environmentally unfriendly boilers and furnaces with modern heating devices, with a preference for those sources that are more environmentally friendly (gas or fuel oil)
2. using renewable energy sources
3. installation of solar systems for the preparation of hot water and in the case of municipal buildings and public buildings—also thermal modernisation. This step is to be implemented where the old heat source was already replaced with a new higher-performance boiler.

When analysing the first of the LERP assumptions, one can unambiguously accept that it is justified and aims to reduce emissions into the atmosphere by replacing old heat sources with high efficiency ones. Of course, a question can be asked about what difference in performance is needed to justify the replacement of a boiler from the ecological point of view. It can be expected that an ecologically justified necessity to replace a boiler with a higher-efficiency one will also depend on the type of fuel used. Unfortunately, there is no clear answer to these questions in the LERP programme. The programmes only indicate the general parameters available during the sale of boilers and indicate the environmental benefits resulting from a direct comparison of atmospheric emissions between these boilers. They do not take into account the environmental load associated with the need to manufacture the new boiler and later with its utilisation.

The analysis of the second part of the first assumption, meaning the replacement of old boilers with more environmentally friendly ones (gas- or oil-fired boilers) and the use of renewable sources of energy, will be carried out later in this article, taking into account the life cycle assessment (LCA) analysis.

The life cycle assessment (LCA) methodology is a method formulated on the basis of two standards: ISO 14040 and ISO 14044. The LCA analysis consists of four essential stages described in the above-mentioned standards (ISO 14040 2006; ISO 14044 2006):

- goal and scope definition
- LCI—life cycle inventory
- LCIA—life cycle impact assessment
- interpretation

LCA is one of several environmental management techniques that is used among others in the study of environmental aspects throughout the life of the product or process, i.e. “from cradle to grave”, which is from extraction of raw materials, through production, distribution, the use phase and reuse or recycling. The LCA technique enables to evaluate the environmental aspects that result from the different life stages of a product. At each stage of product life, as a result of the inventory (LCI), material and energy flows are allocated to individual unit processes restricted by system boundaries. The environmental impact assessment is the result of the evaluation method used (Eco indicator 99 method was applied) and the values of material and energy flows introduced into the computer programme; the above study uses SimaPro computer programme (Sleeswijk 2003; Dylewski and Adamczyk 2011; Dzikuć 2015).

The literature of the subject includes many examples of its application in issues related to the energy or construction industry, e.g. in Dzikuć (2013), Adamczyk and Dzikuć (2014), Lewandowska et al. (2013), Pushkar et al. (2005) and Piwowar et al. (2016).

The environmental impact assessment associated with the change of heat sources for more environmentally friendly ones will be performed using the emission values given in Vieitez and Wolf (2011) and Lachman (2013). The analysis assumes a functional unit equal to 1 kWh of thermal energy production for each heat source. A product system was also adopted—all heat sources are new, and the emission values (Table 6) include the process of the production of thermal energy in the place of its use. The product system does not include the environmental costs associated with the production and recycling of the heat sources (boilers or heat pumps). The emission values are calculated on the basis of primary energy and based on seasonal performance factors. The analysis uses the Eco-indicator 99 assessment method, which is a method commonly used in different sectors of the economy (Dylewski and Adamczyk 2011).

**Table 6:** Values of emissions from different types of heat sources

Emissions → Heat sources ↓	CO_2_ [kg/kWh]	NO_*x*_ [mg/kWh]	CO [mg/kWh]	PM [mg/kWh]	VOCs [mg/kWh]
Oil-fired boilers	0.36	140	50	40	8
Gas boilers	0.22	80	100	9	15
Hybrid heat pump combined with a gas condensing boiler	0.18	90	0	20	10
Heat pumps taking heat from water or ground	0.12	100	0	25	10
Gas absorption heat pumps	0.15	30	30	5	9
Gas (ICE) compressor heat pumps	0.15	95	200	8	12
Coal-fired boiler	0.61	470	1150	280	685
Biomass-fired boiler loaded manually	0.04	80	3200	140	135
Biomass-fired boiler loaded automatically	0.03	65	250	70	40
Pellet-fired boiler	0.07	240	650	40	6
Boiler fired with large pieces of wood	0.02	340	600	52	22
Wood burning fireplace (open combustion chamber)	0.33	290	1600	415	30

Source: Vieitez and Wolf 2011; Lachman 2013

The coal-fired boiler is characterised by the highest environmental impact in terms of carbon dioxide emissions. However, despite high harmfulness of this kind of heat source, not all of the emission types analysed are of the highest impact. A wood burning fireplace emits the highest values of particle matter into the atmosphere.

Based on the emission data (Table 6), it is difficult to clearly identify the heat source with the highest environmental impact; therefore, a proposal was made to conduct an LCA analysis based on the Eco-indicator 99 assessment method and a weighing procedure.

The results of the LCA analysis clearly identify the coal-fired boiler as the source with the highest environmental impact within the presented product system boundaries (3.12 mPt). Its total environmental impact is twice as high as that of the fuel oil boiler (1.55 mPt) (see Table 7). The wood-burning fireplace is also characterised by a high impact on the environment (2.26 mPt). The lowest impact on the environment is that of the boiler fired with large pieces of wood (0.21 mPt).

**Table 7:** The results of the LCA analysis for heat production

Heat sources ↓	Results of the LCA [mPt]
Oil-fired boilers	1.553922
Gas boilers	0.91819
Hybrid heat pump combined with a gas condensing boiler	0.776525
Heat pumps taking heat from water or ground	0.542635
Gas absorption heat pumps	0.622817
Gas (ICE) compressor heat pumps	0.632734
Coal-fired boiler	3.116343
Biomass-fired boiler loaded manually	0.529055
Biomass-fired boiler loaded automatically	0.277351
Pellet-fired boiler	0.384091
Boiler fired with large pieces of wood	0.205086
Wood burning fireplace (open combustion chamber)	2.26401

Therefore, the above-mentioned action associated with the ban on the use of solid fuels for heating purposes in Kraków is completely justified in the light of these results.

In order to determine the optimal solution, a multi-criterion analysis was conducted on selected heat sources. The multi-criterion analysis is a set of algorithms used in the selection of the most advantageous solution for e.g. thermal modernisation projects. Each criterion is assigned a weight of 0.5 (the weighting for a particular group in relation to other groups ranges from 0 to 1). A weight was adopted that treats either criterion at the same level. The lowest values of the two criteria are considered the optimum solution.

The most economically and ecologically optimal heating solution is wood (the boiler fired with large pieces of wood). The differences in the environmental impact for combustion of biomass (wood) are significant (Tables 7 and 8) and depend on the method in which this process is conducted.

**Table 8:** Multi-criterion analysis

Heat source type	Ecological criterion (weight 0.5)	Economic criterion (weight 0.5)
Oil-fired boilers	0.78	52.50
Gas-fired boilers (nitrogen-rich natural gas)	0.46	44.15
Coal-fired boiler	1.56	14.15
Biomass-fired boiler loaded manually	0.27	6.40
Biomass-fired boiler loaded automatically	0.14	6.40
Pellet-fired boiler	0.19	17.50
Boiler fired with large pieces of wood	0.10	6.40
Wood burning fireplace (open combustion chamber)	1.13	6.40

Source: own study based on Tables 2 and 7

It would be very reasonable to take into account the results of the multi-criterion analysis in the LERPs. It should be noted that due to the variability of the energy carrier prices and the technological advances in the design of new boilers, the values of the multi-criterion analysis are subject to constant change. However, the use of the latest knowledge reduces the possibility of mistakes in programming the low emission reduction system. In the LERP programmes being analysed, potential users are recommended to use such a heat source that makes it impossible to burn municipal waste. Burning waste in boilers is a solution that is often used in households. Heat source efficiency and emissions are obviously taken into account. However, if one takes into consideration the economic analysis, the boiler fired with large pieces of wood proves an optimal solution.

## Conclusions

Due to particle matter and benzo(a)pyrene pollution in the air, the Polish economy loses from PLN 40 to 120 billion per year, which includes for example the costs of hospitalisation, lost working days and enterprise losses.

The low emission reduction system in Poland requires legislative changes, as the implementation of the assumptions established in the low emission reduction programmes and the air protection programmes is rather impossible without a support from the state.

### A proposal to use legal and economic instruments to reduce low emission in Poland

Looking for legal solutions conducive to the reduction of the environmental impact of the low emission, a recommendation is made to introduce a tax on pollutants contained in coal, as it is done for individual households in Western European countries. Such a solution would increase the competitiveness of the heat sources with a lower pollutant load emitted into the atmosphere. An alternative to the above-mentioned solution can be changes in tax law pertaining to the periodic reduction of the amount of property tax for the households using an environmentally friendly heat source.

An important element for the improvement of the current low emission reduction system is the introduction of tools of “indirect” stimulation of greener attitudes among owners of residential buildings in the form of preferential loans and/or grants and the use of the funds obtained in this way for a non-refundable support to owners/administrators of individual residential buildings. Actions of this kind allow to reduce the use of residents’ own funds in the investment process, which, in turn, results in the acceleration of the implementation of the projects and achievement of real environmental and energetic benefits.

The low emission reduction programmes assume that as users of the environment, single-family building owners, tenants and managers will be motivated to replace boilers with higher-efficiency and/or greener boilers, a result of the application of the appropriate financial instruments (Adamczyk 2014).

As the experiences of different local government units show, the implementation of the low emission reduction programmes contributes significantly to the improvement of the atmosphere. This is achieved mainly by notifying building owners of the financing sources available for thermal modernisation projects. The building owners see economic benefits in these activities, first and foremost. The implementation of the LERP programmes has a significant impact on the change of primary energy carrier for the purpose of heating buildings—from a solid fuel (hard coal, often of poor quality) to other greener fuels (natural gas, fuel oil or biomass). The fashion in which these fuels are burned is also important, as was shown in the LCA analysis. In addition, this makes it possible to burn energy carriers in a more efficient manner (through the replacement of low-performance boilers and furnaces with high-efficiency units as well as installation of renewable energy sources). The heating equipment efficiency is of vital importance in the context of the environmental impact. The Commission Regulation (EU) 2015/1188 of 28 April 2015, which takes into account Directive of the European Parliament and of the Council 2009/125/EC of 21 October 2009 *laying down general principles for the setting of ecodesign requirements for energy-related products in annex II*, lists the minimum efficiency values for heating devices in force in the European Union as of 1 January 2018 (Commission Regulation (EU) 2015/1188). The transposition of these requirements into the published LERP programmes would facilitate the process of making purchase decisions by property owners even now and would improve the state of the atmosphere.

### A proposal of system changes affecting the reduction of low emission in Poland

An important step for the entire low emission reduction system would be to give greater privileges to chimney-sweeps for the control and supervision of installations in order to ensure good health and safety of residents. Of course, this would require amendments to the legislation in force, as well. In Poland, the chimney-sweep is the only legal person to have immediate contact with individual heat sources and would be able to check these sources in addition to performing chimney installation checks. An alternative to this solution could be the establishment of a separate administrative unit for control or assignment of such privileges to an existing unit e.g. the National Sanitary Inspectorate, which works on the municipal level, following a change of law. The chimney-sweep could also be responsible for evaluating the class of heat source efficiency in individual boiler houses. In the case of an irregularity, they would be obliged to inform users about the need to exchange for higher energy efficiency heat sources (especially for fixed boilers). The Czech Republic introduced a ban on the use of low energy efficiency boilers a few years ago.

### A proposal for educating the public about the harmful effects of burning waste in boilers

All these aspects contribute to the reduction of the emissions of substances harmful to the environment. It should be noted that the indirect effects of the implementation of the programmes include forcing behavioural changes among residents. In the winter (heating period), household furnaces are often used to burn some fractions of municipal waste that should be disposed of in an alternative. This results in an emission of the most hazardous compounds—a process that is difficult to assess. This behaviour is not controlled effectively in Poland, as there are no organisational units tasked with this duty. According to the authors, the use of educational spots in the media could draw public attention to the threat posed by waste incineration in domestic boilers.

The *Polish Energy Policy until 2030* (currently in force) does not include any direct references to “the low emission” as an important risk to the state of the atmosphere (*Polityka energetyczna Polski do 2030 roku* 2009). This may indicate that the past decade did not pay much attention to the above problem. Currently, work is underway to adopt the new *Polish Energy Policy until 2050*, which already includes an appropriate provision that gives much importance to this type of pollution source (*Projekt Polityki Energetycznej Polski do 2050 roku* 2015).

## Footnotes

1 Low emission—the emission of products of the combustion of solid, liquid and gaseous fuels into the atmosphere from the emission sources (emitters) located at the altitude not greater than 40 m. A distinction is made between transport emissions, industrial emissions and emissions from the production of heat for central heating and hot water preparation. The combustion products that affect the low emission include the following gases: CO_2_, CO, SO_2_, NO_*x*_, polycyclic aromatic hydrocarbons, such as benzo(a)pyrene and dioxins, and heavy metals (lead, arsenic, nickel, cadmium) and particulate matter PM_10_ and PM_2.5_ (Kaczmarczyk 2015).

2
World Health Organization

3 In the case of the so-called low emission sources—i.e. households, services, agriculture and others—the emission values are provided for the year 2013 (GUS 2015).

4 The total emission of the main air pollutants encompasses combustion processes in the energy production and transformation sector, non-industrial combustion processes, industrial combustion processes, production processes, fossil fuel mining and distribution, application of solvents and other products, road transportation and waste management.

5 Currency exchange rate: 1 Euro ≈ 4.20 PLN.

6 The health protection assessment was performed for 46 zones: agglomerations, city zones (above 100,000 residents) and zones (the remaining parts of the provinces). The assessments based on the plant protection criteria were performed only for 16 zones (the assessment excluded agglomerations and city zones) (*Rozporządzenie Ministra Środowiska z dnia 2 sierpnia* 2012).

7 Environment Protection Inspection—a two-instance institution subordinate to the minister responsible for the issues of the environment, consisting of the Main Inspectorate for the Environmental Protection and 16 provincial inspectorates

8 The division into zones is made on the basis of the Regulation of the Minister of Environment of 2 August 2012 on the zones where the air quality assessment is performed (*Rozporządzenie Ministra Środowiska z dnia 2 sierpnia* 2012).

9 The inclusion of a zone in class C or B results from exceedances of a certain criterion value used for substance concentration levels on a specific, usually quite limited, area within the zone and should not be equated with poor air quality throughout the zone.

10 Administrative division of Poland: 1st degree—16 provinces, 2nd degree—380 districts, 3rd degree—2479 municipalities
